# Acute Impact of Dietary Pattern and Walking on Postprandial Attention, Mood, and Satiety in Older Adults: A Randomized Crossover Trial

**DOI:** 10.3390/nu11102294

**Published:** 2019-09-26

**Authors:** Christina Diekmann, Michael Wagner, Hanna Huber, Manuela Preuß, Peter Preuß, Hans-Georg Predel, Birgit Stoffel-Wagner, Rolf Fimmers, Peter Stehle, Sarah Egert

**Affiliations:** 1 Department of Nutrition and Food Sciences, Nutritional Physiology, University of Bonn, 53115 Bonn, Germany; 2 Department for Neurodegenerative Diseases and Geriatric Psychiatry, University Hospital Bonn, 53127 Bonn, Germany; 3 DZNE, German Center for Neurodegenerative Diseases, 53127 Bonn, Germany; 4 Human Resource Development, Healthy Campus Bonn, University of Bonn, 53115 Bonn, Germany; 5 University Sports, University of Bonn, 53117 Bonn, Germany; 6 Department of Circulation Research and Sports Medicine, Preventive and Rehabilitative Sports and Performance Medicine, German Sport University Cologne, 50933 Cologne, Germany; 7 Central Laboratory, Institute of Clinical Chemistry and Clinical Pharmacology, University Hospital Bonn, 53127 Bonn, Germany; 8 Institute of Medical Biometry, Informatics and Epidemiology, University Hospital Bonn, 53127 Bonn, Germany; 9 Institute of Nutritional Medicine, University of Hohenheim, 70599 Stuttgart, Germany

**Keywords:** postprandial metabolism, physical activity, walking, postprandial attention, postprandial mood, satiety, hunger, appetite, cortisol

## Abstract

Research suggests that attention, mood, and satiety can be influenced by meal composition and postprandial activity. The present study examined whether this hypothesis applies to persons with a risk phenotype for the development of cardiovascular/neurodegenerative diseases. A randomized crossover trial was conducted in subjects with metabolic syndrome traits (*n* = 26, 8 female, age 70 ± 5, BMI 30.3 ± 2.3 kg/m^2^). Each subject participated in four interventions: iso-energetic (4300 kJ) meals (Western diet high-fat, WD, and Mediterranean-type diet, MD) followed by either 30 min of moderate walking (4.6 ± 0.1 km/h) or rest. Attention, mood, satiety and plasma cortisol concentrations were measured at fasting and 1.5, 3.0, 4.5 h postprandially. Data were analyzed by linear mixed models. In all interventions, attention increased continuously in the postprandial period (time effect, *P* < 0.001). After WD, attention was lower after walking compared to resting (meal × activity effect, *P* < 0.05). Postprandial mood was generally “good” with no intervention effects. Postprandial satiety increased reaching maximum at 1.5 h after meal (time effect, *P* < 0.001) and was higher after MD compared to WD (meal effect, *P* < 0.001). In all interventions, plasma cortisol decreased similar to its diurnal variation (time effect, *P* < 0.001). In our subjects, meal composition had no relevant impact on attention and mood. After typical WD, resting instead of walking seems to have a more beneficial effect on postprandial attention. MD leads to a strong and long-lasting feeling of satiety, possibly resulting in reduced energy intake in the further course of the day and, thus, long-term effect on weight control.

## 1. Introduction

Research suggests that the sedentary lifestyle of today’s Western societies is associated with the development of chronic systemic low-grade inflammation, which is at the root of many typically Western diseases associated with the metabolic syndrome [1]. Due to the anti-inflammatory capacities of aerobic physical activity, integration of regular activity sessions into everyday life seems to be beneficial regarding improvement of general health and, furthermore, regarding protection of the brain from metabolic stress [1,2]. In addition, especially regular aerobic activity has been consistently reported to prevent mental illness (e.g., depression) and alleviate mood problems, as well as to improve cognitive and brain function, but even acute, moderate activity sessions seem to exert similar effects [2,3,4,5]. In this context, different research studies describe modulators of effect size being the temporal sequencing of cognitive assessment in relation to exercise (e.g., following exercise session or during exercise session), the modality of aerobic training (e.g., cycling or running), and the cognitive parameters measured (e.g., memory or processing speed), as well as age and medical condition [3,4,6].

While supplementation studies suggest that single nutrients (e.g., docosahexaenoic acid) can increase cognitive performance, research on the effects of whole meals and, especially, different meal compositions on cognitive function and mood is limited [7]. Compared to a meal intake which is in accordance with the Western dietary pattern, a Mediterranean diet provides higher levels of nutrients including essential fatty acids, vitamins, minerals, and antioxidants, which seem to support brain function. Additionally, the Mediterranean dietary pattern contains fewer refined carbohydrates and saturated fatty acids, which have been associated with cognitive deficits [7,8]. Therefore, it is likely that a regular choice of food items characteristic for the Mediterranean dietary pattern is beneficial with regard to the prevention of age-related cognitive deficits [9,10]. In this context, numerous epidemiological studies suggest that an adherence to plant-based dietary patterns, especially the Mediterranean dietary pattern, is associated with improved cognitive performance, slower age-related cognitive decline and lower risk of cognitive impairment and neurodegenerative disease in older adults [11,12,13,14].

Current randomized interventions trials evaluating the acute interactive effects of meal composition and physical activity on cognitive performance and emotions are limited [15,16] and to the best of our knowledge, no previous human study has investigated the acute effects of postprandial exercise suitable for daily implementation neither on postprandial attention as a complex cognitive function, nor on mood/emotions following the consumption of meals reflecting different dietary patterns, especially in subjects with a risk phenotype for the development of cardiovascular and neurodegenerative diseases (e.g., elevated age; characteristics of metabolic syndrome). Since current research suggests that breakfast as the first meal of the day is most important from a dietary perspective [17] and is vital for optimal cognitive function and intellectual performance by providing readily available energy to the brain [18], the test meals in the present study were provided as breakfast challenges after an overnight fast (≥12 h). The study tested two main hypotheses: (i) a Mediterranean-type diet meal (MD) generates higher postprandial satiety, postprandial attention and a better subjective mood than an iso-energetic Western diet high-fat meal (WD); and (ii) Moderate walking in the postprandial period as compared to remaining sedentary, results in increased postprandial attention and a better subjective mood. In addition to these main hypotheses, this study examined the impact of plasma cortisol concentration on postprandial attention, since elevated systemic cortisol concentrations have been associated with detrimental effects on cognition and a long-term contribution to Alzheimer’s disease pathology [19,20]. In this context, the present study evaluated if the activity session or the intake of high-energy meals were high enough stressors to trigger cortisol release and if effects of different plasma cortisol concentrations on postprandial attention are relevant even in an acute study design.

The data presented in this manuscript are ancillary examinations (secondary outcome measures) of the intervention study which has initially been designed to investigate the effects of meal composition and postmeal walking on different metabolic, inflammatory, oxidative, and endothelial events in the postprandial period, with postprandial triglycerides as primary outcome measure [21].

## 2. Materials and Methods

### 2.1. Participants

Details of the study design and subject recruitment, enrollment, and randomization have been described previously [21]. In brief, interested volunteers (*n* = 31) aged 60 to 80 y attended a screening that included physical assessments (e.g., body height and weight, waist circumference), clinical assessments (e.g., liver and kidney function, serum lipids and lipoproteins, plasma glucose), medical history, and the documentation of dietary habits. Main inclusion criteria were: (i) overweight or obesity stage 1 (BMI 27–34.9 kg/m^2^), (ii) visceral fat distribution (waist circumference ≥ 94 cm for men and ≥80 cm for women), and (iii) prehypertension (systolic blood pressure: ≥120 mmHg, and ≤139 mmHg; diastolic blood pressure: ≥80 mmHg, and ≤89 mmHg) or stage 1 hypertension (systolic blood pressure: ≥140 mmHg, and ≤159 mmHg; diastolic blood pressure: ≥90 mmHg, and ≤99 mmHg). In addition, study eligibility required fulfillment of at least one of the following criteria: (i) dyslipidemia (fasting serum triglycerides ≥ 1.7 mmol/L and/or serum HDL-cholesterol < 1.0 mmol/L for men and <1.3 mmol/L for women), (ii) increased plasma glucose (fasting plasma glucose ≥ 5.6 mmol/L) or (iii) a pro-inflammatory state (hs-CRP ≥ 2.0 mg/L). A total of 26 subjects (18 male, eight female) were included in the study. All subjects completed the entire intervention trial, and their respective data were included in the analysis. The study was conducted in accordance with the guidelines laid down in the 1964 Declaration of Helsinki and its later amendments. All study procedures were approved by the ethics committee of the Medical Faculty of the University of Bonn, Germany (ethic approval code 070/17). Written informed consent was obtained from all subjects prior to inclusion. This trial was registered at http://www.germanctr.de and http://drks.de under identifier DRKS00012409.

### 2.2. Study Design

This postprandial study was conducted as a randomized controlled crossover trial at the Department of Nutrition and Food Sciences, Nutritional Physiology (University of Bonn, 53115 Bonn, Germany). All subjects participated in four treatment conditions, each lasting 4.5 h from morning to afternoon. Study days were separated by 2 wk wash-out periods. The order of treatments was randomized for each subject via computer-generated randomization tables (Microsoft Excel 2010, Microsoft Corp., Redmond, WA., USA). The four treatment conditions were as follows: (i) MD plus 30 min postprandial walking; (ii) MD plus 30 min postprandial resting; (iii) WD plus 30 min postprandial walking and (iv) WD plus 30 min postprandial resting. On each treatment day, venous blood sampling for cortisol analysis and questionnaires for attention, mood, and satiety were taken at fasting (0 h) and at three time points during the postprandial period (1.5, 3.0, and 4.5 h). The outcome measures shown in the present manuscript were examined as secondary outcome measures in the context of the randomized crossover trial evaluating the impact of meal composition and postmeal walking on selected postprandial events. Further details of this trial and the study design have been previously described [21]. 

#### 2.2.1. Test Meal

WD and MD test meals (challenges) were designed specifically for the purposes of the present study. The meals were iso-energetic (4300 kJ/meal) and iso-nitrogenous. WD was rich in total fat, saturated fatty acids, and animal protein. MD was rich in unsaturated fatty acids, dietary fiber, and antioxidative compounds (Table 1). The main components of the WD were croissant, bread roll, jam, butter, cold cuts, boiled egg and cream yogurt. The MD mainly comprised ciabatta, smoked salmon, muesli, fruit, and vegetables. Details of food items and nutrient composition of MD and WD have been previously described [21]. 

Study personnel prepared both breakfast challenges according to a standardized protocol, which included the weighing of each food item to the nearest gram. The test meals were consumed in the morning as first meal of the day and had to be ingested within 20 min.

#### 2.2.2. Physical Activity

The activity (walking or resting) was conducted directly after meal intake. The 30 min walking session was performed outdoors on the University of Bonn campus. Participants were accompanied by study personnel. The duration was selected to reflect current recommendations for the minimum level of moderate-intensity physical activity required on most days of the week to reduce the risk of cardiovascular events [22]. During the first walking session, the subjects were told to walk at a moderate pace, reflecting their individual speed of comfort. For standardization purposes, this speed was then reproduced during the following walking session. The resting (control) phase was conducted at the study location. Each participant remained in a supine position, and was told to abstain from any distraction over a period of 30 min [21]. 

### 2.3. Measurements

#### 2.3.1. Assessment of Cognitive Abilities: Attention/Focus

The German version of the Frankfurt Attention Inventory 2 (FAIR-2, Test version A) was used, which is a validated paper and pencil measure of visual selective attention as one facet of cognitive function with good psychometric properties [23,24] and proven sensitivity to pharmacological interventions [25,26,27]. Test completion requires the accurate and quick identification and marking of target items among similar non-target items. In this context, a continuous line needs to be drawn under each row of items, with a peak clearly pointing into the target-items. Participants need to be alert and show a rapid reaction time, as well as the ability to maintain focused attention throughout the test duration. Both accuracy and speed are taken into account when evaluating test performance and are combined into an overall performance score (K score). The FAIR-2 was conducted in the fasting state (baseline) and 1.5, 3.0, and 4.5 h postprandially on each of the four study days. All subjects were familiarized with FAIR-2 prior to the commencement of the study to reduce practice effects.

#### 2.3.2. Assessment of Subjective Mood

The German version of the Multidimensional Mood State Questionnaire (MDMQ) was used to assess subjective mood [28]. The MDMQ is a paper and pencil test, which uses selected adjectives (24 in the long form and 12 in the short form), which can be assigned to three bipolar dimensions of mood: (i) good mood vs. bad mood, (ii) alertness vs. fatigue, and (iii) ease vs. unease. For each adjective, subjects were asked to classify their current feeling on a five-point scale from 1 (“not at all”) to 5 (“very much”). An overall score was calculated for each of the three bipolar dimensions, with higher (or lower) values associated with the respective positive (or negative) sensations. The test was conducted in the fasting state (baseline) and 1.5, 3.0, and 4.5 h postprandially on each of the four study days. The two versions of the short form (type A and B) were used alternately at the respective time points, with the order being randomized for each subject. All subjects were familiarized with the MDMQ prior to the commencement of the study to reduce practice effects.

#### 2.3.3. Assessment of Hunger, Appetite, and Satiety

The sensations ‘hunger’, ‘appetite’ and ‘satiety’ were assessed using 100-mm visual analogue scales (VAS). On three different VAS, the sensations ‘hunger’, ‘appetite’ and ‘satiety’ were paired with the correspondingly opposite sensations ‘no hunger’, ‘no appetite’ and ‘no satiety’. Subjects were requested to make a vertical mark on each scale that best matched their current feeling. Each score was determined by measuring the distance from the left side of the line to the vertical mark. Since VAS values for hunger and appetite were highly correlated (r = 0.8), both were combined to a value describing the overall sensation of hunger / desire to eat (arithmetic mean). The test was conducted in the fasting state (baseline) and 1.5, 3.0, and 4.5 h postprandially on each of the four study days. All subjects were familiarized with the VAS scales prior to the commencement of the study to reduce practice effects.

### 2.4. Blood Sample Processing and Analysis

Details of the pre-analytical procedures of fasting and postprandial blood samples have been described previously [21]. Fasting and postprandial cortisol was analyzed in duplicate using commercially available enzyme-linked immunoassay kits (IBL International GmbH, Hamburg, Germany) in accordance with the manufacturer’s instructions and recommended quality control procedures.

### 2.5. Sample Size Calculation

The outcomes measured in the present manuscript were examined as secondary outcome measures as part of the randomized crossover trial evaluating the impact of meal composition and postmeal walking on selected postprandial events. Therefore, a priori sample size calculation was based on serum triglycerides as primary outcome measure of the entire postprandial intervention study [21].

### 2.6. Statistical Analysis

All statistical analyses were performed using the IBM SPSS statistical software package (SPSS version 25, IBM Corporation, Somers, NY, USA). The linear mixed model procedure was used to test the effects of meal and activity, time points, and their interaction on postprandial cortisol and FAIR-2, MDMQ, and VAS values. Meal type (WD, MD); activity (walking, resting); time points (1.5, 3.0, 4.5 h postprandially); and interactions (meal × activity, meal × time, activity × time, and meal × activity × time) were set as fixed factors and subject identifier as a random factor. Baseline values were included as covariates. For FAIR-2 analysis, the order of study visits was set as an additional covariate in order to control for any residual practice effects. In all tests, the residuals showed no relevant deviations from a normal distribution. In case of significant meal × activity interaction, the linear mixed model procedure was performed separately for WD and MD to examine possible activity effects for both meal types. Furthermore, incremental area under the curve (iAUC) was calculated for all parameters using the trapezoidal rule, and the linear mixed model procedure was performed to test for meal, activity, and meal × activity effects. In all analyses, significance level was set at *P* < 0.05. Descriptive data are presented as arithmetic mean ± standard error of the mean (SEM), unless otherwise stated.

## 3. Results

### 3.1. Participants

Baseline characteristics are shown in Table 2. All participants were overweight (46.2%) or obese (53.8%), had a visceral fat distribution, and were prehypertensive or displayed stage 1 hypertension [21].

### 3.2. FAIR-2

In all four treatment conditions, the overall test performance (K score) increased over time (*P* < 0.001) which was due to an increase in speed (*P* < 0.001) rather than an increase in accuracy (*P* = 0.429) over time.

**Figure 1:**
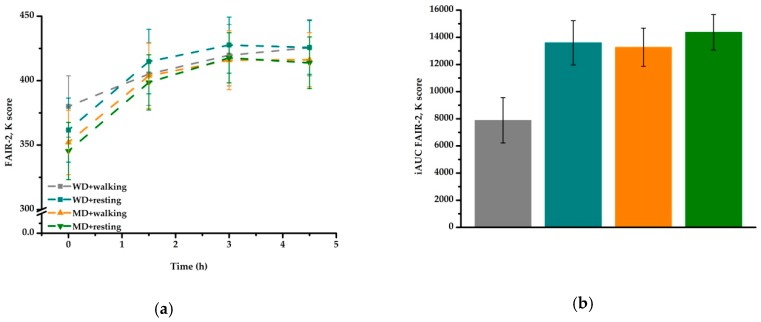
Fasting and postprandial attention (FAIR-2, K score) according to treatment condition: (**a**) K score over time: the overall test performance increased over time (*P* < 0.001). A significant interaction for meal × activity (*P* = 0.026) was observed: for WD but not MD, values were lower after walking compared to resting (*P* < 0.001) (**b**) iAUC of K score: data revealed an effect of meal type (*P* = 0.045) and activity (*P* = 0.027). FAIR-2, Frankfurt Attention Inventory 2; iAUC, incremental area under the curve; MD, Mediterranean-type diet meal; WD, Western diet high-fat meal.

A significant interaction for meal × activity (*P* = 0.026) was observed: for WD but not MD, K scores were lower after walking compared to resting (*P* < 0.001) (Figure 1a, Table 3). Comparison of iAUC data revealed an effect of meal type (*P* = 0.045) and activity (*P* = 0.027), with higher K scores for MD compared to WD and for resting compared to walking (Figure 1b).

### 3.3. MDMQ

For the values of the dimension good vs. bad mood, a time effect was observed (*P* = 0.014), as well as a significant interaction for meal × activity (*P* = 0.027). Here, an activity effect was observed for MD (*P* = 0.004) but not for WD. The values for the dimension alertness vs. fatigue were significantly influenced by time (*P* = 0.006) and meal (*P* = 0.003), with lower values after WD compared to MD. The values for the dimension ease vs. unease were affected by activity (*P* = 0.022) but neither by time nor by meal type. For all three dimensions, neither a meal × time nor an activity × time effect has been observed. Comparison of iAUC data revealed no effect for meal or activity either. Data are summarized in Table 4. 

### 3.4. VAS

**Figure 2:**
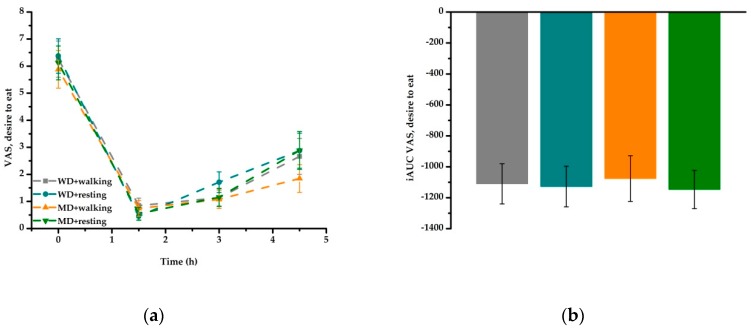
Fasting and postprandial hunger (desire to eat) according to treatment condition: (**a**) the overall sensation of hunger decreased over time (*P* < 0.001) in all four treatment conditions. (**b**) iAUC revealed no meal or activity effect. iAUC, incremental area under the curve; MD, Mediterranean-type diet meal; VAS, visual analogue scale; WD, Western diet high-fat meal.

**Figure 3:**
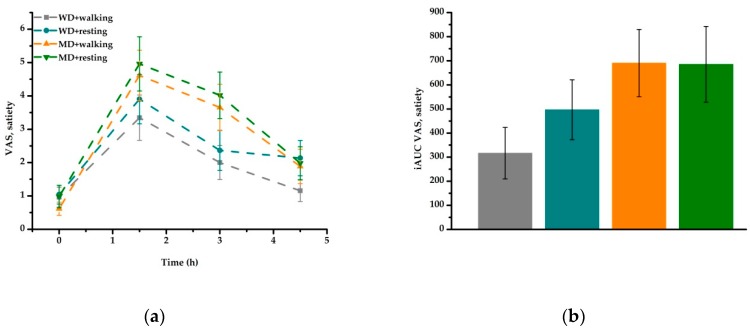
Fasting and postprandial satiety according to treatment condition: (**a**) satiety increased over time (*P* < 0.001) with higher values for MD compared to WD (*P* < 0.001). (**b**) iAUC data confirmed the observed meal effect (*P* = 0.004). iAUC, incremental area under the curve; MD, Mediterranean-type diet meal; VAS, visual analogue scale; WD, Western diet high-fat meal.

The overall sensation of hunger (desire to eat) decreased over time (*P* < 0.001), with no meal or activity effect (Figure 2a, Table 5). Comparison of iAUC data confirmed these results (Figure 2b). Satiety values increased over time (*P* < 0.001) with higher values for MD compared to WD (*P* < 0.001) (Figure 3a, Table 5). This meal effect was confirmed by the iAUC data (Figure 3b).

### 3.5. Plasma Cortisol

**Figure 4:**
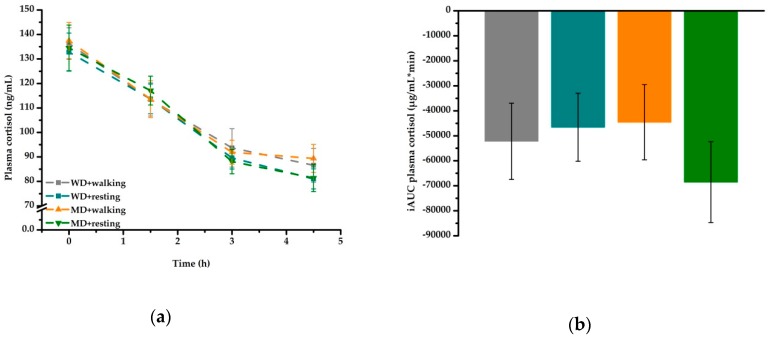
Fasting and postprandial plasma cortisol according to treatment condition: (**a**) postprandial cortisol concentration decreased over time (*P* < 0.001) in all four treatment conditions. (**b**) iAUC data revealed no effect of meal or activity. iAUC, incremental area under the curve; MD, Mediterranean-type diet meal; WD, Western diet high-fat meal.

Postprandial cortisol concentration decreased over time (*P* < 0.001), with no effect being observed for meal or activity (Figure 4a, Table 6). Comparison of iAUC data confirmed these results. Data are summarized in Figure 4b. 

## 4. Discussion

The purpose of this study was to investigate the acute impact of dietary composition and moderate postmeal walking on postprandial attention (representing one aspect of cognitive function), mood, and the sensations of hunger and satiety in older subjects with a risk phenotype for the development of cardiovascular and neurodegenerative diseases.

Prior research suggests that the consumption of breakfast is vital for optimal cognitive performance, since it provides readily available energy as first meal of the day after an overnight fast [15,18]. In the present study, the consumption of both test meals (breakfast challenges) yielded a reliable and substantial (10–20%) increase of postprandial attention over time. iAUC data revealed that the increase in postprandial attention was significantly higher after MD than after WD, which is in accordance with our hypothesis that a MD results in higher postprandial attention than a WD. Considering the fact that glucose is the main fuel for brain function and therefore plays an important role in general cognitive performance [29,30], the observed meal effect on postprandial attention in the present study might be due to the higher amount of carbohydrate in MD (133.3 g vs. 93.7 g). It remains unclear whether the different nutrient composition of the breakfast challenges (Table 1) contributed to the observed results. Since an adherence to the Mediterranean dietary pattern is in the long run associated with improved cognitive performance and mental health, future intervention studies in this field of research might consider taking into account the habitual diet/nutritional status of their participants as a further possible influencing factor of acute nutritional effects on cognitive parameters such as postprandial attention. 

Furthermore, an activity effect was observed after WD, with lower postprandial attention after walking than after resting. From the perspective of energy availability to the brain, these findings are rather unexpected, since we observed higher plasma glucose concentrations after walking than after resting for both meal types [21].

An increase in postprandial cortisol concentrations could further explain the decreased attention values observed after the WD + walking treatment, since elevated plasma cortisol is associated with altered cognitive function [31]. However, our data showed no activity effect on postprandial plasma cortisol over time for none of the four treatments. Since the walking session in the present study was of moderate intensity and short duration only, the stressor might not have been high enough to trigger an increase in plasma cortisol concentrations [32]. However, it is possible that an activity effect on postprandial cortisol would have been detectable if blood samples had been taken during and/or directly at the end of the walking session [32]. In accordance with previous postprandial trials [33,34], in the present study, postprandial cortisol significantly decreased from morning to afternoon, similar to its diurnal variation, and the stimulus of meal intake was not strong enough to alter this distinct pattern (Figure 4a, Table 6). Since postprandial attention as well as cortisol showed a strong time effect, it is difficult to separate between the extent to which time influenced postprandial attention and the extent to which postprandial cortisol had an influence on this parameter. 

The fact that the increase of overall postprandial attention was due to an increase in test speed and not in test accuracy suggests, that the repetition of the same test procedure on each time point on each of the four study days led to a learning effect. This learning effect, however, cannot be quantified but should be taken in to account when evaluating the practical relevance of the statistical results.

In contrast to a previous postprandial exercise trial conducted in healthy, habitually active, middle-aged women, which showed an association between the consumption of breakfast and lower fatigue and higher overall mood and alertness post-exercise [16], in the present study, no relevant effect of meal intake or postprandial activity behavior on the measured mood dimensions (good vs. bad mood, alertness vs. fatigue, and ease vs. unease) could be observed (Table 4). Despite statistical significance, for all three dimensions, changes in mean values between all time points were minor. The obtained results showed that the subjects were alert, at ease and in rather good mood during the course of each study day (Table 4). Since meal or activity effects on subjective parameters like mood are complex to measure, it is possible that the MDMQ was not sensitive enough to determine clinically relevant treatment effects on mood over time [35]. 

In comparison to the present study, future intervention studies in this field of research might consider the habitual activity level/physical activity status of their participants as a modulator of effect size. It can be assumed that the intensity of an acute physical activity session is perceived differently depending on the activity status of a person (low or high metabolic stressor), possibly resulting in different effects on postprandial attention and / or postprandial emotional state. 

Research suggests that the consumption of food in the early morning leads to control and moderation of total energy intake throughout the whole day, since the complex carbohydrates usually consumed during breakfast affect activity as well as release of hormones (e.g., gastric inhibitory peptide, glucagon-like peptide-1, cholecystokinin), which differently affect postprandial plasma glucose and, consequently, satiety [18]. In the present study, the higher satiety values over time that were observed after MD treatments compared to WD treatments are mainly due to the higher volume of the MD (781 g/MD meal vs. 390 g/WD meal), as well as the higher dietary fiber content (14.5 g/MD meal vs. 4.2 g/WD meal). It is possible that this marked satiating power led to control and moderation of total energy intake throughout the remaining day.

The major strengths of the present study are the controlled, crossover design, the absence of study dropouts, and the high rate of treatment compliance. In contrast to previous studies in this flied of research, this trial followed a holistic approach and focused on regular meals reflecting different dietary patterns and not on the administration of nutrient solutions (e.g., fat tolerance tests). The 30 min session of moderate postmeal walking was designed to be easily incorporable into daily routines, even by inactive or physically more restricted individuals. One limitation of the present work is that it is an explorative analysis of secondary outcome measures. The study was originally designed to investigate the effects of meal composition and postmeal walking on different metabolic, inflammatory, oxidative, and endothelial outcome measures in the postprandial period and a priori sample size calculation was based on serum triglycerides as primary outcome measure of the postprandial intervention study [21]. Another potential limitation of the study is the time points selected for the measurement of outcome measures, which may not have been representative of the overall postprandial period. However, for scheduling reasons, further time points and shorter intervals were precluded from the study design. Furthermore, the repeated use of the FAIR-2 type A to examine attention behavior may have led to a learning effect which might have influenced time- and treatment effects. However, alternate use of both test types (A and B) could not be performed as there was a risk of possible bias due to changes in target items and test procedures. Future intervention trials focusing on the evaluation of selective attention as a primary outcome measure might consider including a variety of test procedures focusing on different aspects of cognitive performance (including vigilance) and a chronometric approach might be reasonable in order to evaluate cognitive function more specifically. This might be especially relevant in chronic rather than acute intervention trials. 

## 5. Conclusions

In conclusion, the present study shows no relevant effect of meal composition or postprandial activity behavior regarding subjective mood and none of the four treatment conditions can be rated superior in older adults with a risk phenotype for cardiovascular diseases. Compared to a Western diet high-fat meal, a meal composition reflecting the Mediterranean dietary patter seems to be beneficial regarding postprandial attention. After the consumption of WD, postprandial resting seems to be more beneficial than postprandial walking for optimal cognitive performance. Due to its nutrient composition and food items (e.g., higher amount of low-energy/nutrient-dense foods and higher fiber content), MD leads to a stronger and longer lasting feeling of satiety. A selection of food items in accordance with the Mediterranean dietary pattern might therefore have a positive impact on weight regulation and should be of special importance in overweight-to-obese subjects. Future randomized, controlled trials should focus on the investigation of the chronic rather than the acute impact of dietary composition and postprandial activity behavior on cognition and emotion to further understand mechanisms involved and to develop strategies to attenuate or even prevent comorbid neurological conditions in risk subjects.

## Figures and Tables

**Table 1:** Nutrient composition per serving of the two breakfast challenges (adapted from [21]).

Energy and Nutrients	WD	MD
Energy (kJ)	4247	4251
Protein (g)	26.1	25.9
Carbohydrates (g)	93.7	133
Dietary fiber (g)	4.2	14.5
Total fat (g)	59.4	40.1
SFAs (g)	32.0	5.1
MUFAs + PUFAs (g)	23.8	30.9
ß-carotene (mg)	0.2	5.4
Vitamin E^1^ (mg)	2.3	12.1
Vitamin C (mg)	9	102

^1^ α-Tocopherol equivalent. MD, Mediterranean-type diet meal; MUFAs, monounsaturated fatty acids; PUFAs, polyunsaturated fatty acids; SFAs, saturated fatty acids; WD, Western diet high-fat meal.

**Table 2:** Baseline characteristics of participants (adapted from [21]) ^1^.

Parameters	Total (*n* = 26)
Age (y)	69.9 ± 4.7
BMI (kg/m^2^)	30.3 ± 2.3
Body fat (%)	38.4 ± 8.1
Waist circumference (cm)	104 ± 5.8
Systolic blood pressure (mmHg)	149 ± 16.4
Diastolic blood pressure (mmHg)	88.3 ± 7.3
Serum triglycerides (mmol/L)	1.76 ± 0.79
Serum total cholesterol (mmol/L)	4.86 ± 1.60
Serum HDL cholesterol (mmol/L)	1.61 ± 0.37
Serum LDL cholesterol (mmol/L)	3.19 ± 0.76
Plasma glucose (mmol/L)	5.64 ± 0.66
Serum hs-CRP (mg/L)	2.5 ± 3.0

^1^ Shown as mean ± SD. BMI, body mass index; HDL, high-density lipoprotein; hs-CRP, high sensitive C-reactive protein; LDL, low-density lipoprotein.

**Table 3:** Fasting and postprandial attention (FAIR-2, K score)^1,2^.

Treatment Conditions	Fasting	Postprandial			*P*-Values from Linear Mixed Models					
	0 h	1.5 h	3.0 h	4.5 h	Time	Meal	Activity	Meal × Activity	Meal × Time	Activity × Time
K score (FAIR-2)					<0.001	0.039	0.001	0.026	0.905	0.770
WD + walking	380 ± 23.8	405 ± 24.3	420 ± 23.9	426 ± 21.8						
WD + resting	362 ± 24.9	415 ± 25.0	428 ± 21.7	426 ± 20.8						
MD + walking	352 ± 25.0	404 ± 25.4	416 ± 22.8	416 ± 21.0						
MD + resting	345 ± 22.3	399 ± 21.4	418 ± 19.4	414 ± 20.1						

^1^ shown as mean ± SEM. *P*-value for meal × time × activity interaction not significant. ^2^ K score describes overall test performance during FAIR-2. Scores range from 0–640. FAIR-2, Frankfurt Attention Inventory 2; MD, Mediterranean-type diet meal; WD, Western diet high-fat meal.

**Table 4:** Fasting and postprandial parameters of MDMQ^1,2.^

Treatment Conditions	Fasting	Postprandial			*P*-Values from Linear Mixed Models					
	0 h	1.5 h	3.0 h	4.5 h	Time	Meal	Activity	Meal × Activity	Meal × Time	Activity × Time
Good vs. bad mood					0.014	0.047	0.171	0.027	0.580	0.804
WD + walking	17.4 ± 0.63	18.5 ± 0.41	18.0 ± 0.45	18.4 ± 0.35						
WD + resting	18.6 ± 0.37	18.7 ± 0.32	18.2 ± 0.35	18.2 ± 0.38						
MD + walking	17.9 ± 0.48	18.0 ± 0.36	17.7 ± 0.45	17.8 ± 0.34						
MD + resting	17.7 ± 0.54	18.1 ± 0.36	18.0 ± 0.40	18.6 ± 0.35						
Alertness vs. fatigue					0.006	0.003	0.089	0.404	0.600	0.573
WD + walking	15.2 ± 0.79	16.4 ± 0.68	15.2 ± 0.65	14.8 ± 0.65						
WD + resting	16.7 ± 0.57	16.3 ± 0.51	16.2 ± 0.56	15.5 ± 0.67						
MD + walking	15.2 ± 0.85	14.9 ± 0.60	14.6 ± 0.71	14.2 ± 0.77						
MD + resting	15.4 ± 0.85	15.8 ± 0.60	14.4 ± 0.75	15.4 ± 0.63						
Ease vs. unease					0.444	0.107	0.022	0.248	0.253	0.837
WD + walking	17.3 ± 0.68	17.6 ± 0.53	17.8 ± 0.53	18.1 ± 0.41						
WD + resting	18.1 ± 0.49	18.4 ± 0.39	18.2 ± 0.45	18.0 ± 0.41						
MD + walking	17.2 ± 0.51	17.8 ± 0.45	17.1 ± 0.61	17.2 ± 0.51						
MD + resting	17.5 ± 0.57	18.0 ± 0.44	17.8 ± 0.49	18.0 ± 0.51						

^1^ shown as mean ± SEM. *P*-value for meal × time × activity interaction not significant. ^2^ values range from 5 (lower end of the scale) until 20 (upper end of the scale). MD, Mediterranean-type diet meal; MDMQ, Multidimensional Mood Sate Questionnaire; WD, Western diet high-fat meal.

**Table 5:** Fasting and postprandial hunger and satiety^1,2.^

Treatment Conditions	Fasting	Postprandial			*P*-Values from Linear Mixed Models					
	0 h	1.5 h	3.0 h	4.5 h	Time	Meal	Activity	Meal × Activity	Meal × Time	Activity × Time
Desire to eat (hunger)					<0.001	0.283	0.359	0.552	0.656	0.105
WD + walking	6.26 ± 0.67	0.85 ± 0.28	1.13 ± 0.32	2.66 ± 0.67						
WD + resting	6.38 ± 0.64	0.45 ± 0.15	1.71 ± 0.38	2.88 ± 0.65						
MD + walking	5.88 ± 0.70	0.74 ± 0.29	1.08 ± 0.34	1.85 ± 0.51						
MD + resting	6.12 ± 0.62	0.55 ± 0.23	1.15 ± 0.33	2.88 ± 0.70						
Satiety					<0.001	<0.001	0.158	0.490	0.160	0.972
WD + walking	0.96 ± 0.30	3.35 ± 0.68	2.00 ± 0.51	1.15 ± 0.32						
WD + resting	1.04 ± 0.28	3.90 ± 0.74	2.37 ± 0.60	2.13 ± 0.53						
MD + walking	0.62 ± 0.20	4.62 ± 0.75	3.65 ± 0.70	1.88 ± 0.52						
MD + resting	0.98 ± 0.34	4.96 ± 0.81	4.02 ± 0.70	1.98 ± 0.49						

^1^ shown as mean ± SEM. *P*-value for meal × time × activity interaction not significant. ^2^ values were measured via VAS and range from 0 (lower end of the scale) until 10 (upper end of the scale). MD, Mediterranean-type diet meal; VAS, visual analogue scale; WD, Western diet high-fat meal.

**Table 6:** Fasting and postprandial plasma cortisol concentration^1.^

Treatment Conditions	Fasting	Postprandial			*P*-Values from Linear Mixed Models					
	0 h	1.5 h	3.0 h	4.5 h	Time	Meal	Activity	Meal × Activity	Meal × Time	Activity × Time
Plasma cortisol (ng/mL)					<0.001	0.810	0.160	0.863	0.659	0.569
WD + walking	136 ± 6.46	114 ± 6.76	93.7 ± 7.86	86.6 ± 6.90						
WD + resting	133 ± 7.76	114 ± 6.07	89.6 ± 4.52	81.0 ± 4.10						
MD + walking	137 ± 7.38	114 ± 7.47	91.9 ± 4.90	89.4 ± 5.71						
MD + resting	134 ± 9.34	117 ± 5.90	88.1 ± 4.96	81.4 ± 5.55						

^1^ shown as mean ± SEM. *P*-value for meal × time × activity interaction not significant. MD, Mediterranean-type diet meal; WD, Western diet high-fat meal.
